# Supplementary material for: Nanosilver–Biopolymer–Silica Composites: Preparation, and Structural and Adsorption Analysis with Evaluation of Antimicrobial Properties
Source: Int J Mol Sci. 2024 Dec 18;25(24):13548. doi: 10.3390/ijms252413548 (PMC11679571; doi:10.3390/ijms252413548)
Supplement: Supplementary file 1 [file ijms-25-13548-s001.zip › ijms-3317120-supplementary.pdf]

SUPPORTING INFORMATION

Nanosilver–Biopolymer–Silica Composites: Preparation, and Structural and Adsorption Analysis with Evaluation of Antimicrobial Properties

Magdalena Blachnio [1,*], Malgorzata Zienkiewicz-Strzalka [1], Jolanta Kutkowska [2] and Anna Derylo-Marczewska [1]

[1] Department of Physical Chemistry, Institute of Chemical Sciences, Maria Curie-Sklodowska University,
Maria Curie-Sklodowska Square 3, 20-031 Lublin, Poland; malgorzata.zienkiewicz-strzalka@mail.umcs.pl (M.Z.-S.); anna.derylo-marczewska@mail.umcs.pl (A.D.-M.)
[2] Department of Genetics and Microbiology, Institute of Biological Sciences, Maria Curie-Sklodowska University, 19 Akademicka Street, 20-033 Lublin, Poland; jolanta.kutkowska@mail.umcs.pl
* Correspondence: magdalena.blachnio@mail.umcs.pl; Tel.: +48-0815375637

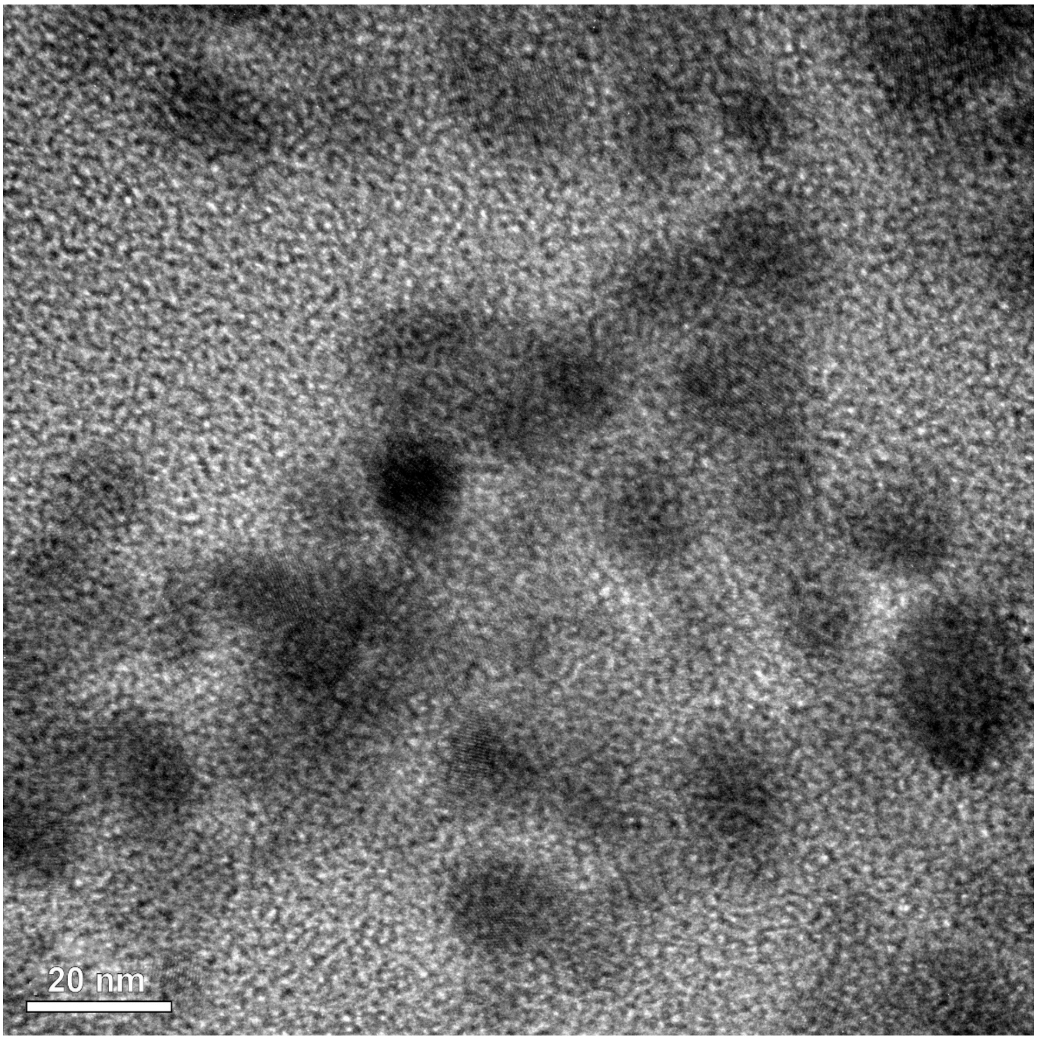

Figure S1. TEM image of initial AgNP solution.

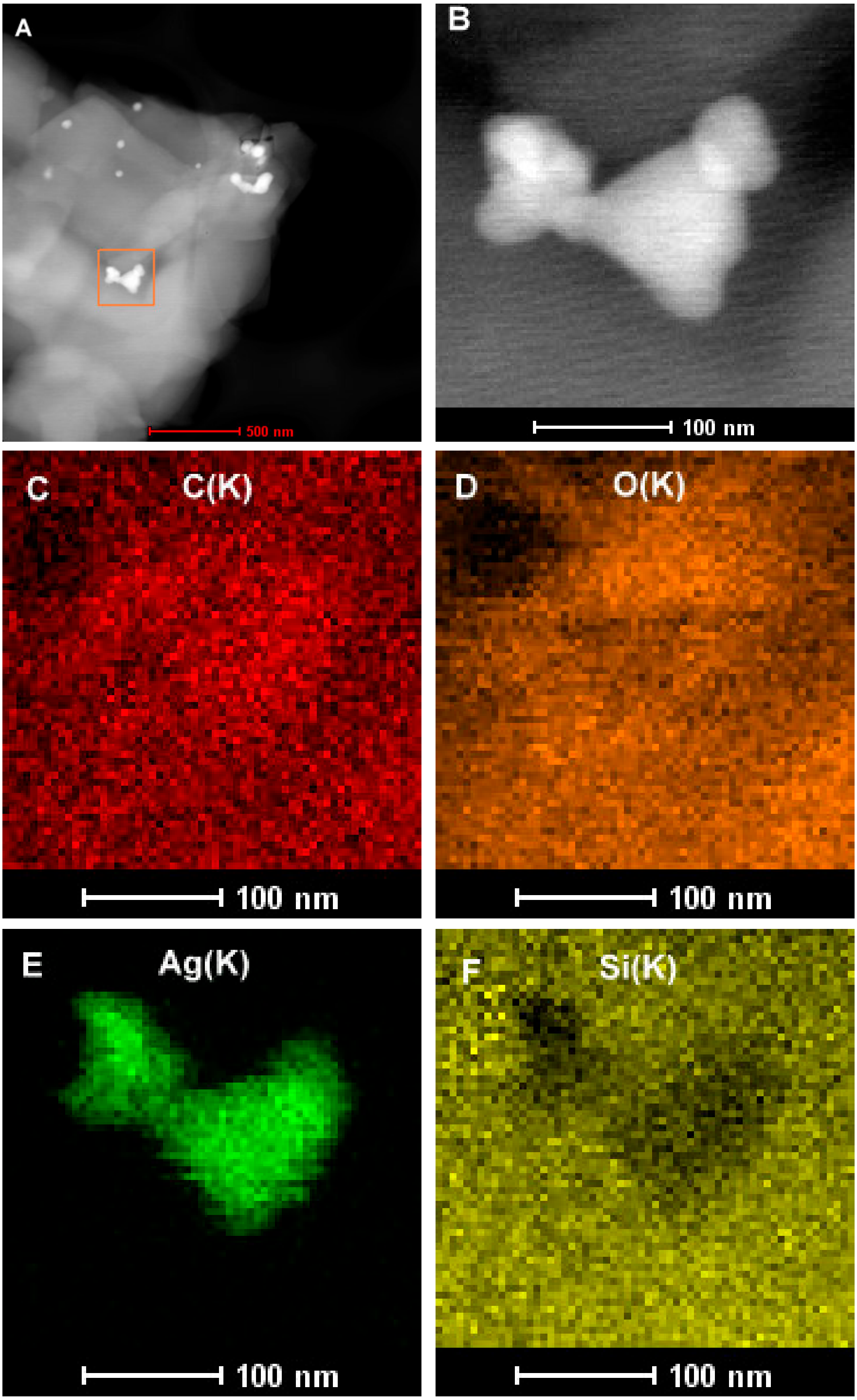

Figure S2. TEM image with energy-dispersive X-ray (EDX) mapping for AgChS3 for different elements (C, O, Ag, Si)
